# Post-Translational Modification as an Allosteric Switch in Hsp90: How Dual Phosphorylation Locks Chaperone Complexes into Hyperstabilized States

**DOI:** 10.1021/acs.jpclett.6c01824

**Published:** 2026-07-06

**Authors:** Giorgio Bonollo, Benedetto Roncati, Luca Torielli, Shujuan Wang, Chiranjeevi Pasala, Silvia Pavoni, Francesco Frigerio, Fabrizio Cinquini, Gabriela Chiosis, Stefano A. Serapian, Giorgio Colombo

**Affiliations:** † Department of Chemistry, 19001 University of Pavia, Via Taramelli 12, 27100, Pavia, Italy; ‡ Chemical Biology Program, 5803 Memorial Sloan Kettering Cancer Center, New York, New York 10065, United States; § Department of Physical Chemistry, R&D EniSpA, Via Felice Maritano 26, 20097 San Donato Milanese (Mi), Italy

## Abstract

Multimicrosecond MD simulations reveal that dual phosphorylation at Ser226/Ser255 of Hsp90β acts as a molecular clamp, rigidifying the overall structure, propagating allosteric coordination changes to distal domains, and stabilizing cochaperone–client interfaces. These findings provide an atomistic mechanism by which post-translational modifications can stabilize Hsp90 interaction states that are compatible with epichaperome formation, with implications for disease biology and therapeutic targeting.

## Graphical abstract

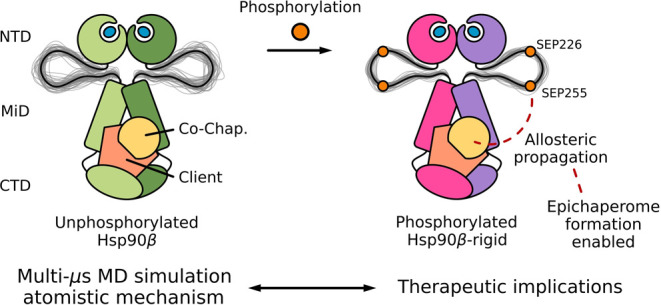

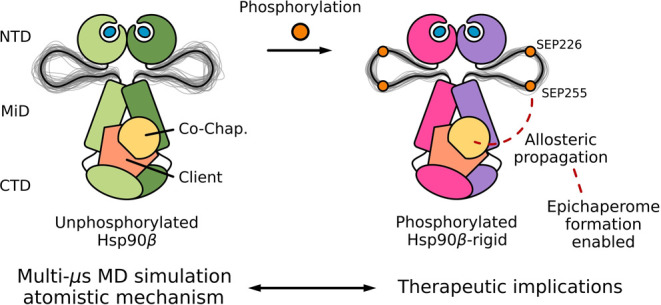

Heat shock protein 90 (Hsp90) is a conserved ATP-dependent molecular chaperone that oversees the folding, activation, and assembly of a broad client repertoire, which ranges from kinases to hormone receptors and transcription factors, making it a central node connecting multiple signaling pathways in the cell. Hsp90 functions as a homodimer: each protomer is composed of three structurally distinct domains: an N-terminal domain (NTD) hosting the ATP-binding pocket, a middle domain (MiD) constituting the principal client- and cochaperone-binding surface, and a C-terminal dimerization domain carrying the MEEVD motif for TPR-cochaperone recruitment. An intrinsically disordered, highly charged linker connecting NTD and MiD serves as an adaptable regulatory scaffold.^1^ Under physiological conditions, Hsp90 cycles through open, semiclosed, and closed conformational states, with sequential ATP hydrolysis—modulated by cochaperones such as Aha1 and p23—driving client processing. In disease contexts, including cancer and neurodegeneration, aberrant post-translational modifications can shift Hsp90 from a dynamic chaperone-cycle component into a more stabilized interaction state compatible with higher-order scaffold formation.^2,3^ In this state, Hsp90 becomes incorporated into epichaperomes, disease-associated supramolecular scaffolding platforms that reorganize protein–protein interaction networks and sustain pathological signaling and survival programs.

Post-Translational Modifications (PTMs) have been shown to provide a mechanistic bridge between stress signals and Hsp90 rewiring in terms of structure, dynamics and function.^4^ Phosphorylation, catalyzed by kinases, introduces a dianionic phosphate that can establish new electrostatic contacts and hydrogen bonds, effectively acting as *molecular glue.* Among the documented Hsp90 phosphorylation events, modification of Ser226 and Ser255 within the charged linker/NTD–MiD regulatory region of Hsp90β has been reported in cancer and neurodegenerative disease contexts. Prior biochemical, chemical-biology, and computational studies identified phosphorylation of Hsp90β Ser226/Ser255, and the corresponding Hsp90α Ser231/Ser263 sites, as PTMs enriched in epichaperome-associated Hsp90 and functionally linked these modifications to HSP90 incorporation into epichaperomes.^5,6^ In that work, phosphomimetic modeling within an HSP90–HSP70–HOP epichaperome-core assembly suggested that charged-linker phosphorylation restricts local flexibility, promotes coordinated assembly motions, and stabilizes Hsp90-containing higher-order assemblies.^5^ However, because those simulations used phosphomimetic substitutions and focused on the epichaperome-core assembly, the atomistic consequences of bona fide dual phosphorylation on Hsp90 conformational states, interdomain allostery, and client/cochaperone interfaces remain unresolved.

Here, we address this gap using multimicrosecond all-atom MD simulations of WT and explicitly dual-phosphorylated HSP90β in semiclosed, closed, and Hsp90–Cdc37–CDK4-bound states. This approach allows us to define how pSer226/pSer255 create local electrostatic contact networks, alter conformational sampling, and propagate allosteric stabilization to distal functional interfaces. Together, these results support a model in which PTMs drive the switch of Hsp90 and its complexes from being flexible and dynamic entities in the WT to more rigid and preorganized states. The latter can aptly support the scaffolding role of Hsp90 in the assembly of multicomponent platforms responsible for pathological functions.

To progress on this route, we carried out multimicrosecond all-atom MD simulations for six systems: isolated WT and doubly phosphorylated (pSer226/pSer255) Hsp90 in the semiclosed state, the closed state, and WT and doubly phosphorylated Hsp90 in the tripartite Hsp90–Cdc37–CDK4 complex^7,8^ (states labeled as *sc*, *cl* and *co* and variants as *WT* and *PH*).

To identify modifications in the short-range interactions determined by the phosphoserine groups, we first performed a hydrogen bond scan, selecting as acceptors the oxygen atoms of serine residues (Oγ and OPs in the case of phosphorylation) and as donors nitrogen atoms of charged residues (Arg and Lys). Next, we applied a formal H-bond criterion (donor–acceptor distance ≤4Å; angle ≥135°), retaining only bonds that resulted to be persistent for ≥50 consecutive simulation frames, equivalent to 2.5 ns (snapshots are in fact saved every 50 ps). WT interactions that met this criterion in any condition were consistently lower than in phosphorylated systems. (Figure 1).

**Figure 1:**
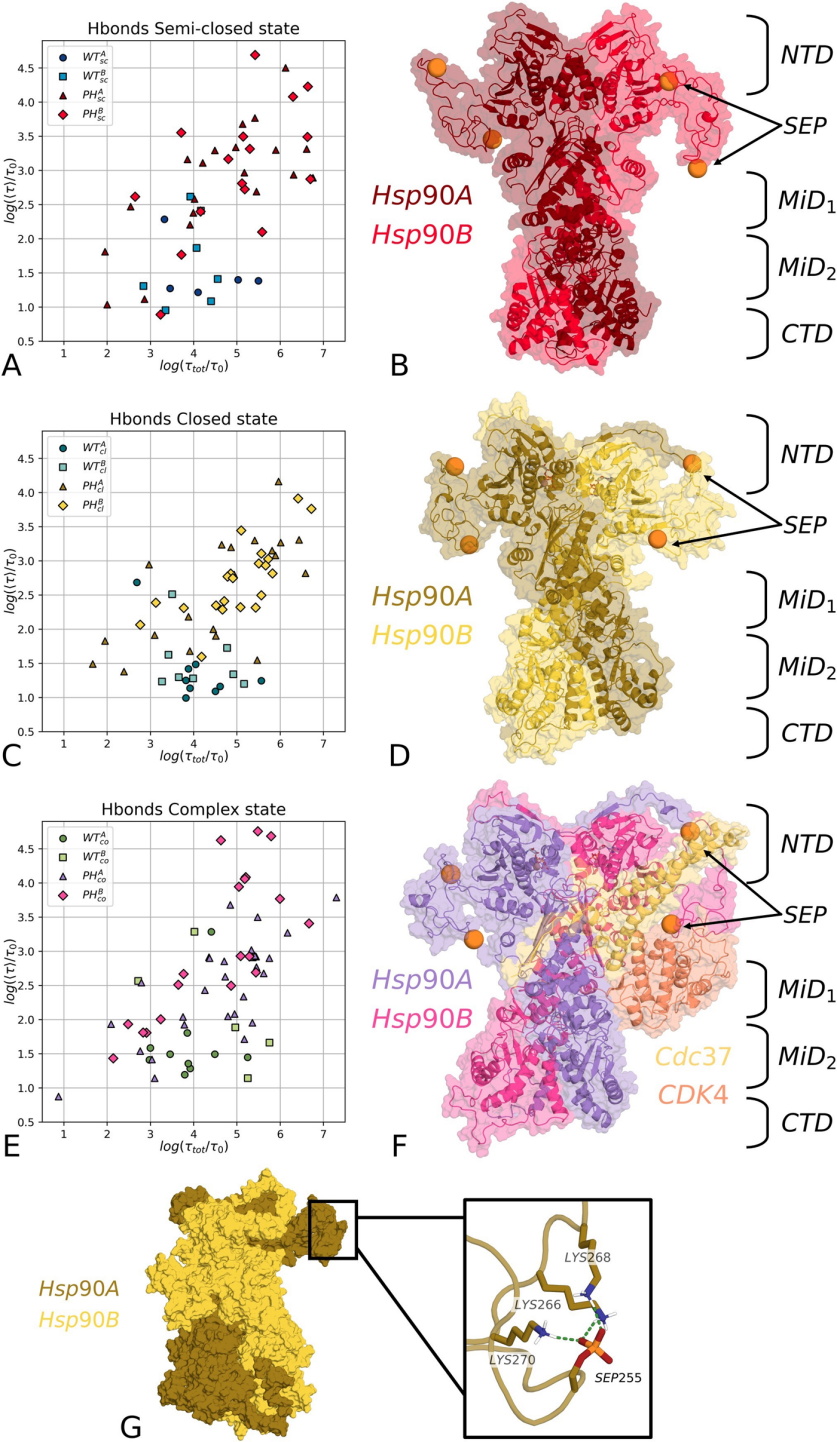
H-bond analysis. On the *x*-axis is a measure related to the total lifetime, while on the *y*-axis is a measure of the average lifetime of each hydrogen bond: (A,C,E) Hydrogen bonds in the Semi-Closed, Closed and Complex states respectively, with different colors and markers differentiating between WT/Phosphorylated state and A/B protomer, (B,D,F) Structures of Semi-Closed, Closed and Complex states, with phosphorylation sites evidenced with orange spheres, (G) Representative frame with SEP226 A exhibiting three H-bonds.

To quantify persistence, we defined two complementary metrics: the average bond lifetime ⟨τ⟩ (mean duration of individual bonding episodes) and the total bond lifetime τ_TOT_ (total number of frames in which the bond is formed, see ESI). To better compare different results, we plotted log­(τ_TOT_/τ_0_) (*x*-axis) versus log­(⟨τ⟩/τ_0_) (*y*-axis) for each hydrogen-bond pair (Figure 1); this allows discrimination between frequent-but-transient interactions (high τ_TOT_, low ⟨τ⟩) and rare-but-durable contacts (high ⟨τ⟩). Durable contacts are more likely to exert structural and functional consequences.

Compared with WT, phosphorylation consistently introduces additional and more persistent hydrogen-bonding interactions, supporting the idea that the dianionic phosphate groups nucleate new local contact networks. Furthermore, a subset of bonds is common to all three phosphorylated systems—most prominently phosphorylated S255_A_–R196_A_—suggesting these represent particularly favorable interactions that persist regardless of overall conformational state. Individual systems may be nonetheless characterized by specific dominant interactions: in the closed state, the SEP226_B_–R221_B_ bond persists for more than one-quarter of the total simulation time, marking it an exceptionally stable interdomain bridge. In the phosphorylated version of the trimeric complex, several hydrogen bonds involve residues belonging to Cdc37 or CDK4, with the S255_B_ – R288_CDK4_ contact emerging as one of the most enduring interactions across all simulated systems.

Next, we computed probability-density-normalized Cα–Cα distance histograms for the residue pairs corresponding to the hydrogen bond with the longest τ_TOT_ for each serine in the phosphorylated case, comparing it to the same pair in the WT case (Figure S1). In these plots, solid lines denote the S226 residues and dashed lines the S255 residue, with different color coding across panels representing distinct monomers in different structural states. In all three Hsp90 states, phosphorylation sharpens the peaks of the Cα–Cα distance distributions, shifting it to shorter values relative to WT, indicating both a reduction in mean separation and a narrowing of the distance variance. This behavior reverberates a mechanism whereby the phosphate group draws positively charged side chains into a tighter, less mobile coordination shell, significantly limiting the sampling of extended loop configurations.

Finally, we computed radial distribution functions g­(r) describing the probability density of Nζ­(Lys)/Cζ­(Arg) atoms as a function of distance r from each phosphoserine Oγ. The cumulative coordination number N­(r), i.e. the running integral of g­(r), provides the average number of basic atoms within radius r. Each panel overlays the phosphorylated and WT curves for direct comparison (Figure S2). In the six panels (two monomers × three conformational states), the phosphorylated g­(r) curves display taller first-shell peaks shifted to shorter r compared to WT, while the WT curves are broader and lower. The cumulative N­(r) rises more steeply for the phosphorylated systems, indicating a higher density of basic atoms at short-range. These results confirm that the phosphate groups create a compact, enriched coordination state, absent in the WT systems. Our data suggest a model in which phosphorylation acts as an electrostatic “molecular clamp”. Such model is confirmed by the observation of distinct Sodium ion distributions around the loop in different conditions (Figure S3).

The structural importance of S226 and S255 phosphorylation prompted us to ask whether cancer-associated mutations map onto these sites, their flanking residues, or their key interaction partners. Mining the COSMIC database (gene HSP90AB1_ENST00000620073) revealed that S226 and S255 themselves are never mutated in cancer, consistent with strong selective pressure to preserve these phosphorylation-competent residues.

In contrast, their immediate neighbors are mutational sites. Around S226, conservative (I225V), charge-inverting (E224K), and charge-neutralizing (D228N) substitutions are documented; around S255, an analogous pattern emerges (D256A/V, G258D/G, E251K). Residues mediating persistent local contacts with the phosphorylated Serines—D264V, K265N, K267N, K268N—show the same trend.

Importantly, R196 and R221, which engage S226/S255 phosphate groups through longer-range, conformationally restrictive contacts, are likewise mutation-free—while the adjacent R197 can mutate to W, Q, or L. This juxtaposition suggests a **coupled mutational behavior**: positions essential for establishing or receiving the phospho-driven interaction network are conserved, whereas their immediate surroundings tolerate—and even favor—substitutions toward hydrophobic or hydrogen-bonding character.

Together, these findings support a model in which S226/S255 phosphorylation, and its long-range electrostatic readout by R196/R221, is functionally indispensable for cancer cell fitness, with surrounding chemical complementarity evolving to reinforce this regulatory interaction.

To determine whether the observed modifications in contact networks impact the internal dynamics of the Semi-Closed, Closed and Complexed states of Hsp90 and whether local loop modifications propagate to distal regions, we computed pairwise distance-fluctuation (DF) matrices for all six systems. DF quantifies the degree of coordination between all residue pairs in a system.^9,10^ Regions/substructures with smaller fluctuations indicate tighter dynamic coupling, even if they may be distal in the primary/tertiary structures. To provide a compact view of the dynamic differences induced by PTMs, we report ΔDF = DF_PH_ – DF_WT_ matrices (Figure S4): positive values (red) indicate that the WT system exhibits greater coordination (lower fluctuation) than the phosphorylated one; negative values (black) indicate the reverse. The results reveal a complex, state-dependent allosteric response to PTMs that could be summarized as follows.

Hsp90 domains were labeled as NTD, MiD and CTD (N-Terminal Domain, Middle Domain and C-Terminal Domain respectively); in some analyses the MiD domain was split into MiD_1_ and MiD_2_ domains (see Figure 1 for structural reference of domains). In the NTD regions, we observed minimal inter-NTD coordination changes across states, with the notable exception of protomer B active site in the closed and complex systems, where phosphorylation increases distal coordination, indicating that phosphorylation may affect ATP processing, modifying Hsp90 cycle and its functional activities. Looking at MiD/CTD–NTD coupling, in the semiclosed and closed states, phosphorylation reduces MiD/CTD coordination with both NTDs, suggesting that modifications in the loop decouples the middle domain from the nucleotide-binding machinery. In the complex, this trend reverses, with a widespread increase in NTD–(MiD/CTD) coordination. Focusing on intra-MiD/CTD and interprotomer MiD/CTD, we observe a minor loss of coordination in semiclosed and closed forms versus a gain of internal coordination in the complex, consistent with assembly dependent stabilization. Interestingly, the Loop regions appear to be the ones with the most state-specific behavior. In the semiclosed form, loop A (on protomer A) loses coordination while loop B (on protomer B) gains it. In the closed form, loop A gains coordination with loop B’s MiD/CTD context while losing it elsewhere, and loop B generally gains coordination. In the complex, loop A partially loses coordination with the majority of the complex while loop B, loses coordination in the interaction with cochaperone and client. Finally, analysis of the Hsp90β–Cdc37/CDK4 interface shows that phosphorylation produces a global enhanced increase in Hsp90β coordination with both Cdc37 and CDK4, consistent with stabilization of the trimeric assembly.

The matrix region reporting on the interaction between loops and the complex is particularly revealing: loop A’s phosphorylation most strongly coordinates client CDK4, providing a structural rationale for how PTMs at Ser226/Ser255 could selectively stabilize the kinase-competent assembly.

These results are corroborated by the study of the volume sampled by loops (Figure 2), showing how phosphorylation impacts also on the motion of unstructured regions. Phosphorylation renders loop A more globular, while loop B becomes more adherent on the NTD surface with respect to the WT case. When interacting with the cochaperone-client system, loop A displays a more localized volume, suggesting a stronger interaction with the client, while loop B more often reaches the lumen region hosting the unstructured tail of the client, suggesting possible interference with the folding mechanism of the client.

**Figure 2:**
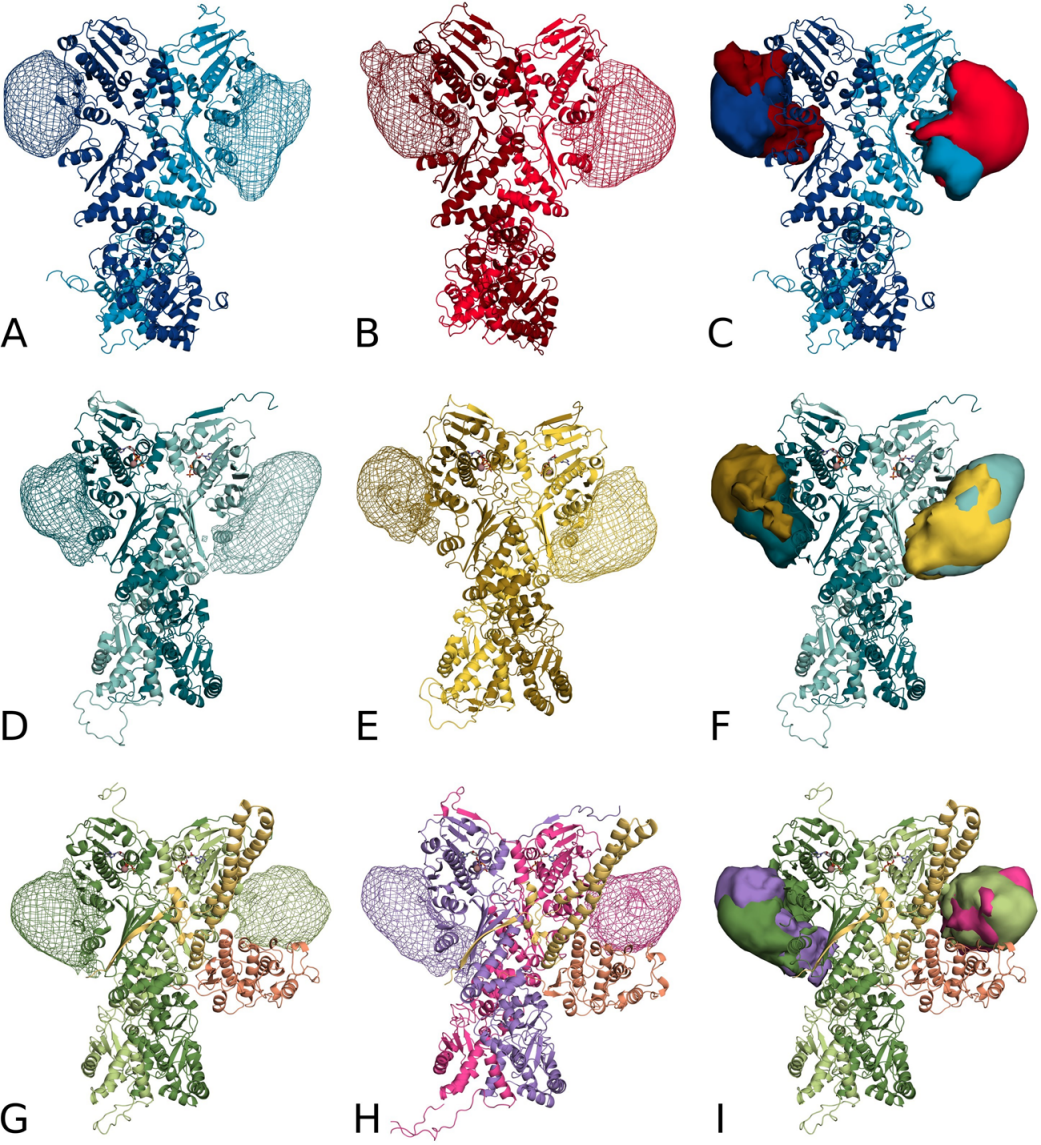
Loop volume changes after phosphorylation. Results of grid calculations: (A) Volume spanned by charged loops in simulations of the Semiclosed WT variant, (B) Volume spanned by charged loops in simulations of the Semiclosed phosphorylated variant, (C) Comparison between volumes spanned by charged loops in simulations of the Semiclosed state between WT and phosphorylated variants, with the phosphorylated structure superimposed onto the WT one and not displayed for clarity, (D) Volume spanned by charged loops in simulations of the Closed WT variant, (E) Volume spanned by charged loops in simulations of the Closed phosphorylated variant, (F) Comparison between volumes spanned by charged loops in simulations of the Closed state between WT and phosphorylated variants, with the phosphorylated structure superimposed onto the WT one and not displayed for clarity, (G) Volume spanned by charged loops in simulations of the Complex WT variant, (H) Volume spanned by charged loops in simulations of the Complex phosphorylated variant, (I) Comparison between volumes spanned by charged loops in simulations of the Complex state between WT and phosphorylated variants, with the phosphorylated structure superimposed onto the WT one and not displayed for clarity.

To investigate the impact of the changes in internal dynamics brought about by phosphorylation on collective motions, we performed tensor of gyration (TOG)^9^ analysis followed by principal component analysis (PCA) on all six trajectories (Figures S5 and S6). The results show that the identity of principal motions is not well preserved, and the trajectory projections on the Principal Components indicate that WT systems explore substantially larger regions of the PC1–PC2 conformational spaces, while phosphorylated systems remain confined to narrow clusters. This reduction in conformational sampling is consistent with the enhanced coordination and hydrogen-bond stability quantified above, confirming phosphorylation as a global switch that locks Hsp90 in a restricted subset of conformations and causes Hsp90 complexes to lose their dynamic character. Next, we mapped the PC directions onto the two physics-based collective variable that define the twisting-closure movement around the lumen, further proved our hypothesis of restricted conformational motion in the Phosphorylated system (Figure 3).

**Figure 3:**
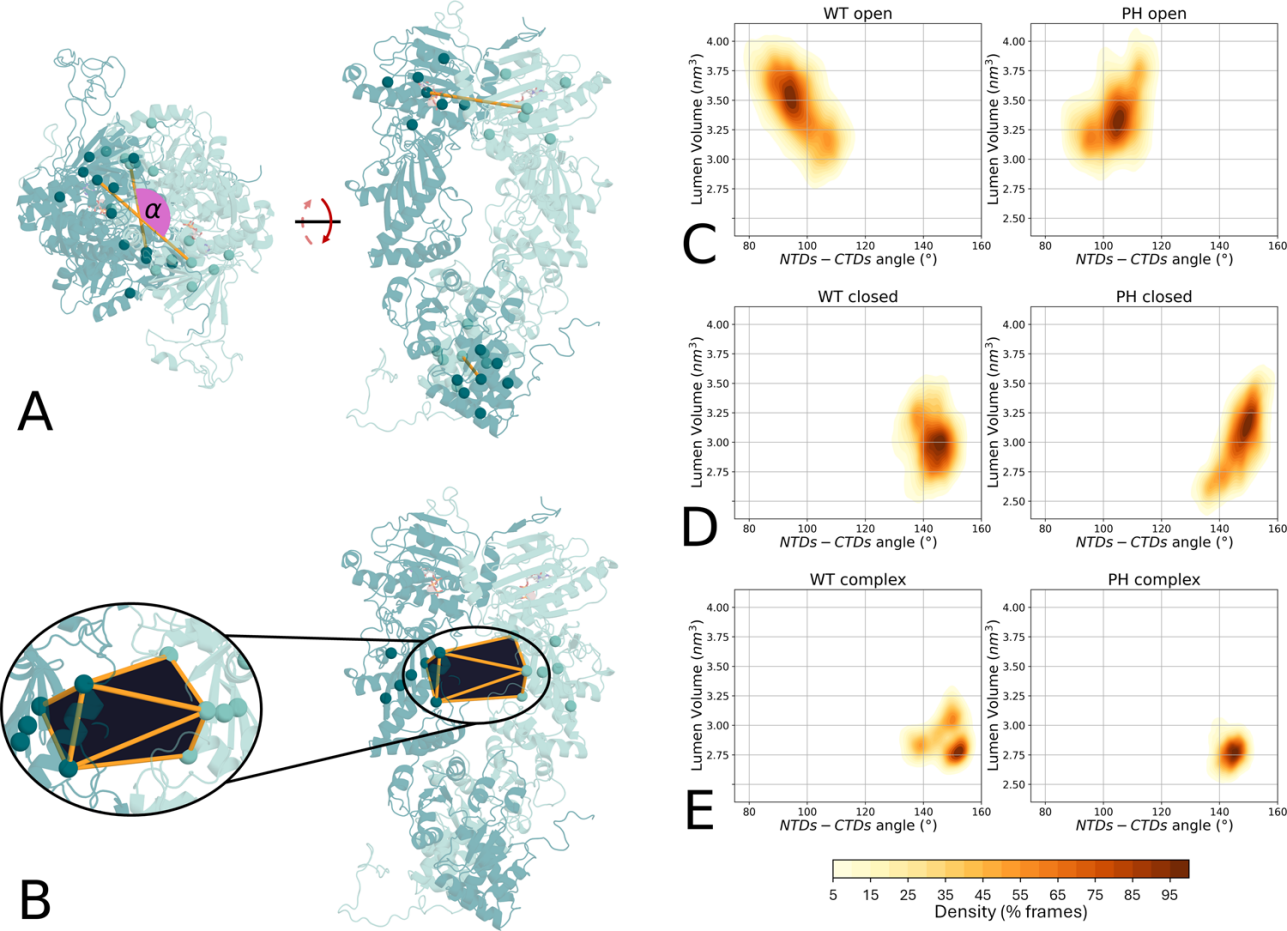
Collective variables capturing twisting-closure motion of Hsp90. (A) The first CV is the angle between NTDs and CTDs connecting vectors (twisting), (B) The second CV is the volume of Hsp90 lumen (triangulation of TOG points facing lumen), (C–E) KDE comparison plot of distribution of CVs in the Semi-Closed, Closed and Complex state.

In conclusion, MD simulations reveal how phosphorylation at Ser226 and Ser255 of Hsp90 mechanistically alters its structure, complexes and thus emerging functions. The dianionic phosphate groups act as molecular clamps, anchoring to nearby Arg and Lys residues and rigidifying the central loop. Normal Hsp90 complexes are dynamic and plastic, enabling rapid assembly and disassembly to favor client hand-over among the various complexes on the folding pathway. Phosphorylation opposes this by locking conformations and stiffening the complex. These local changes propagate allosterically to distal domains, including the middle domain (MiD) and, in the trimeric complex, to cochaperone Cdc37 and client kinase CDK4. Indeed, cross-chain contacts suggest direct reinforcement of CDK4 engagement by protomer B. In isolated phosphorylated Hsp90, reduced NTD–MiD/CTD coordination may impair ATPase-coupled conformational cycling, favoring longer-lived interaction states compatible with epichaperome scaffold assembly.^6,11,12^ In the full complex, phosphorylation enhances global coordination, indicating that the modified assembly dynamics could impair the release of the complex. In this model, post-translational modifications can shape the functional outcome of chaperone activities with a complex spectrum of impacts. We also note that the two protomers respond asymmetrically, consistent with the known alternating cooperativity of the Hsp90 ATPase cycle, meaning identical phosphorylation events can produce nonequivalent effects.

The present study was designed around the biologically observed dual-phosphorylated state. Based on prior experimental data,^5^ both S226 and S255 phosphorylation are enriched in epichaperome-associated HSP90, so the dual state is the disease-relevant configuration. However, the structural positions could point to nonequivalent contributions: S226 likely promotes/nucleates linker reorientation, whereas S255 likely stabilizes the reoriented linker and the client/cochaperone-bound assembly. Future single-site simulations or phospho-specific mutants would be needed to formally resolve monosite sufficiency.

One may ask at this point whether these observations are limited to CDK4 chaperoning, or may generalize to other kinases (and even different client families). Because Hsp90 engages a broad clientele of oncogenic kinases through expectedly structurally analogous Cdc37-bridged ternary complexes, we hypothesize that phosphorylation-driven rigidification at sites homologous to Ser226/Ser255 may represent a generalizable strategy for tuning client retention across the kinome. Many Hsp90–Cdc37–kinase assemblies share conserved interfacial architecture and similar dependence on NTD–MiD–CTD conformational coupling (also observed for other clients),^13^ suggesting that analogous PTM clamps could analogously bias the chaperone cycle toward long-lived, epichaperome-compatible states for kinase clients beyond CDK4, including other CMGC- and CDK-family kinases reliant on Cdc37 for folding and stability. This raises the possibility that PTM-encoded conformational control is a broadly conserved and diffuse layer of chaperone-mediated oncogenic signaling.

Therapeutically, shedding light on the mechanisms that rewire cellular circuitries and support the emergence of complex supramolecular functional assemblies may hold key implications for both disease understanding and drug design.^14,15^ Therapeutic agents capable of selectively engaging the epichaperome state or the dynamic states that support it, rather than the outright inhibition of chaperone ATPase, could deliver improved selectivity and novel chemotypes. In conclusion, our work shows, at atomistic resolution, how Hsp90 PTMs determine structural and dynamic perturbations that may translate in the emergence of new functional profiles at large scales.

## Supplementary Material



## Data Availability

The analysis tools used in this paper are available from github/colombolab. Data for running simulations are available at: 10.5281/zenodo.20141897.

## Notes

Extensive detailed description of system modeling, simulation setup, and supplementary analyses (PDF)
