# Supplementary material for: Post-Translational Modification as an Allosteric Switch in Hsp90: How Dual Phosphorylation Locks Chaperone Complexes into Hyperstabilized States
Source: J Phys Chem Lett. 2026 Jul 6;17(28):8098–104. doi: 10.1021/acs.jpclett.6c01824 (PMC13383824; doi:10.1021/acs.jpclett.6c01824)
Supplement: Supplementary file 1 [file jz6c01824_si_001.pdf]

Supplementary Information for

Post-Translational Modification as an Allosteric Switch in Hsp90: How Dual Phosphorylation Locks Chaperone Complexes into Hyperstabilized States

Giorgio Bonollo,[a] Benedetto Roncati,[a] Luca Torielli,[a] Shujuan Wang,[b] Chiranjeevi Pasala,[b] Silvia Pavoni,[c] Francesco Frigerio,[c] Fabrizio Cinquini,[c] Gabriela Chiosis,[b] Stefano A. Serapian,[a] Giorgio Colombo[a,*]

Department of Chemistry, University of Pavia, Pavia (Italy).
Chemical Biology Program, Memorial Sloan Kettering Cancer Center, New York, NY 10065, USA.
Department of Physical Chemistry, R&D EniSpA, San Donato Milanese (Mi), Italy
* Corresponding author: g.colombo@unipv.it.

This file contains the supplementary information regarding simulations of Hsp90β protein in three states (namely Semi-Closed, Closed and Complex states, also referred to as *sc*, *cl* and *co* states respectively) in Wild-Type (WT) form and Phosphorylated (PH) form with four phosphorylated sites (two phosphorylated serines, S226 and S255 in each monomer).

1. System modeling

To study of how the different conformational states of Hsp90β are affected by phosphorylated serines, different structures of Hsp90 were selected as initial templates. The first state to be modeled was the Closed Wild-Type state (initial structure kindly provided by Prof. David A. Agard, UCSF), which served as template for the other states. Such complex is resolved from residue 2 to 219 and from residue 274 to 724. Residue 1 was added with the PyMol package [1], while to generate a model for the loop on protomer A, the MODELLER package was employed by initially producing 25 models using the DOPE-HR (DOPE-High-Resolution) statistical potential [2, 3].

Each model was then refined applying up to 300 steps of conjugate gradient minimization to increasingly large residue intervals in each of the 25 models. Then, a predefined “slow” MD annealing procedure sets in, in which each model is heated from 0 K to 1000 K over a total of 1100 4 fs MD steps, followed by cooling back to 300 K over 4800 steps. This procedure was repeated for two cycles.

After the procedure, MODELLER-generated models mod01 to mod25 were assessed in terms of two standard MODELLER scores: DOPE and molecular probability density function. Both scores concurred in identifying model mod04 as the best generated and refined model, thus it was chosen to only continue with mod04 as loop model. An identical copy of protomer A loop was incorporated also in protomer B, thus obtaining the initial Wild-Type Closed state structure.

To model the Semi-Closed state structure, PDB entry 7kw7 was utilized as a structural reference. This structure features the Hsp90α isoform in a Semi-Closed conformation, bound to three cochaperones (two Hsp70 molecules and one Hsp70/Hsp90 Organizing Protein Hop), as well as to a client (Glucocorticoid receptor). All proteins apart from Hsp90 were removed from the complex, and the different aminoacids were mutated with PyMol from the Hsp90α sequence to obtain the Hsp90β isoform. Then, missing residues in the structure were modeled by superimposing the complete Closed structure—using the backbone atoms of the three residues immediately preceding and following the gap—to the equivalent atoms in the Semi-Closed state. Namely, 3 loops were pasted into each Hsp90 protomer (the N-Terminal Domain, NTD, initial loop, residues 1-15, the charged linker, residues 219-274, and the C-Terminal Domain, CTD, terminal loop, residues 700-724).

To model the Complex state structure, PDB entry 5fwk was utilized, where the Hsp90β isoform is present bound to a cochaperone (Cdc37) and to a client (Cdk4). To model the missing residues, the Closed state

structure model was utilized in a similar fashion as done for the Open state, while in this case the sequence did not require any mutations, since the desired isoform was already present.
The three systems were then processed with *reduce* utility from *AmberTools* (v. 2021) which added hydrogen atoms and elaborated likely histidine tautomerization states and likely sidechain orientations in histidines, asparagines, glutamines. Subsequently, protonation states for each system were assessed with the propKa package (v. 3.1) and kept equal between phosphorylated and Wild-Type structure.
Prior to phosphorylation (if applicable) and solvation, a preliminary round of structural minimization in vacuo was carried out on the different HSP90β models using the parmed utility. The sole purpose of this preminimization was to relax those areas in protomer A and protomer B that are adjacent to 'anchor points' in the starting structure onto which we pasted different loop models, especially in the Open and Complex state where also initial and final loops were pasted.
After obtaining the minimized Wild-Type structures, the serine residues in which we were interested in were phosphorylated with the PyMol package (namely, substituting the hydrogen at Oγ of serines 226 and 255 of each protomer with a phosphate group $-PO_3^{2-}$ and renaming it as *SEP*).
Crystallographic waters coming from high resolution X-ray crystal structures of the Hsp90β N-Terminal Domain (PDB entry 6n8y) and Middle Domain (PDB entry 3pry) were retained by superimposing these structures to the same domain of each Hsp90 protomer in the starting models.
The six modeled systems were then solvated in isometric truncated octahedron boxes with TIP3P water molecules, imposing the minimum distance between the solvated protein and one edge of the box to be of at least 11.5 Å, and neutralized by the addition of sodium counterions.

2. Simulations

After solvation, MD simulations underwent a pre-production procedure to properly equilibrate the system. Protein molecules were described with the *ff14SBphosphoaa* forcefield and water with the *TIP3P* model [4, 5]. Sodium counterions were treated with Joung and Cheatham parameters [6], while Mg^{2+} ions were treated with Allnér and Nilsson parameters [7], and the ATP nucleotide present in the Closed and Complex states was parameterized according to Meagher and coworkers.

Simulations were carried out with the AMBER molecular simulation suite [8]. The pre-production procedure consisted of different steps: minimization, solvent equilibration, heating, and system equilibration. Minimization consisted in two step run with *Sander*: a first minimization restrained protein heavy atoms with a harmonic force constant of 5 kcal mol^{-1} $Å^{-2}$, allowing hydrogens and solvent atoms to move (300 minimization steps, 10 with steepest descent algorithm and 290 of conjugate gradient). The second minimization was performed with the same algorithm but without any positional restraints, *i.e.,* also allowing movements of heavy atoms.

The solvent equilibration stage is needed to remove the intrinsic order of water molecules inserted into the system in the solvation step. In detail, 9 ps of MD simulation were performed with a timestep of 1 fs in the NVT ensemble, restraining non water atoms with a force constant of 10 kcal mol^{-1} $Å^{-2}$. This stage was divided into three steps, a first heating 25 K to 400 K in the first 3 ps, followed by a constant 400 K stage, with a final lowering of the temperature from 400 K to 300 K. The temperature coupling was kept fixed at 0.2 ps for the first two steps and decreased from 2.0 ps to 1.0 ps in the final step.

After solvent equilibration, the system experiences a 20 ps heating stage from 25 K to 300 K with a 2 fs timestep in the NVT ensemble with Langevin thermostat [9, 10]. Backbone Cα atoms were restrained with a 5 kcal mol^{-1} $Å^{-2}$ harmonic constant to limit backbone conformations, and SHAKE algorithm [11] was imposed on all bonds containing hydrogen atoms. At the beginning of this stage velocities were reassigned differently for each replica, ensuring a different conformational sampling among replicas.

Equilibration consisted of four steps, with progressively relaxed atomic restraints; all the four steps were simulated in the NpT ensemble with Langevin thermostat and Berendsen barostat [12] (at 300 K and 1 atm), with 2 fs timestep and the SHAKE algorithm on hydrogen-containing bonds. In the first step the simulation lasted 20 ps with Cα atoms positionally restrained with a 3.5 kcal mol^{-1} $Å^{-2}$ harmonic constant, temperature coupling γ_{LN}=1 and pressure coupling constant of 1.0 ps. Restraints were gradually relaxed to 1.75 kcal mol^{-1} $Å^{-2}$ in the second step, and entirely lifted in the third step, which lasted 1 ns. The last step was again 1 ns long and the Langevin constant was increased to 5.

Production run setup was equal to the last step of equilibration (NpT ensemble, 300 K, Langevin thermostat with γ_{LN} coupling set to 5, 1 atm with pressure coupling of 1.0 ps with Berendsen barostat and 2 fs of timestep) and lasted 1 μs for each replica; snapshots were saved every 50ps. A cutoff of 8 Å was applied for the calculation of Lennard-Jones and Coulomb interactions in the direct space, and the Particle Mesh Ewald method [13] was applied to calculate the sole Coulomb interactions beyond this cutoff. The SHAKE algorithm was applied, again, to constrain all bonds involving hydrogen atoms.

For the energy minimization and solvent equilibration steps, CPU-based calculations with *sander* were employed, while the GPU-accelerated *pmemd* was used to run the following stages.

Trajectory Analysis

3. Hydrogen bond analysis

To analyse the differences induced by the phosphorylation in different states we firstly investigated the chemical differences in the proximity of the phosphorylated serine residues. To do that, we mapped all possible hydrogen bonds between Wild-Type or phosphorylated serine oxygens as acceptors (acc) and nitrogens of positively charged residues (namely, lysines and arginines) as donors (don).

More specifically, with the *hbond* command in the *cpptraj* suite we monitored the presence of bonds between $\left[O_{\gamma}^{SER}\right]_{acc} - \left[N_{\zeta}^{LYS}, N_{\varepsilon}^{ARG}, N_{\eta 1}^{ARG}, N_{\eta 2}^{ARG}\right]_{don}$ in the Wild-Type case and bonds $\left[O_{\gamma}^{SEP}, O_{\delta 1}^{SEP}, O_{\delta 2}^{SEP}, O_{\delta 3}^{SEP}\right]_{acc} - \left[N_{\zeta}^{LYS}, N_{\varepsilon}^{ARG}, N_{\eta 1}^{ARG}, N_{\eta 2}^{ARG}\right]_{don}$ in the phosphorylated case. Indeed, the presence of an additional negative charge and additional acceptors on the phosphorylated serine sidechain is expected to increase hydrogen bonding events. Since in this type of analysis we are interested in the local effect induced by the phosphorylation, we monitored hydrogen bond formation during the entire trajectory.

To quantitatively evaluate the differences between the two cases we retrieved two quantities: total hydrogen bond lifetime τ_{TOT}, which is the total number of frames in which the hydrogen bond is detected, and the average lifetime $\langle\tau\rangle$, that is the average number of consecutive frames for which a binding event survives. The occurrence of a hydrogen bond formation is defined by a geometric criterion based on distance and angle threshold. For this analysis we set the maximum distance threshold between the donor and the acceptor to be $max\big(dist(don - acc)\big) = 4\text{Å}$ and the minimum angle threshold between donor, hydrogen donor and acceptor to be $min\big(angle(don - H_{don} - acc)\big) = 135°$. Moreover, we mitigated the effects of intermittency smoothening the calculation of average lifetime with a procedure explained below. Indeed, the transient nature of hydrogen bonds could interfere with the statistical significance of the average lifetime since fast disruption-formation events could lead to many intervals of just a few frames, rather than—as one would expect—fewer intervals with a longer lifetime. Then, we artificially considered a hydrogen bond as being formed also in those frames where geometric criterions were not satisfied but that were less than 20 frames away from bonding events. This enabled us to join separate intervals in which the bond was formed but were separated by a few frames, essentially considering them to be joint intervals.

At the end of the analysis, we retrieved the i bonds that satisfied the conditions:

$$\tau_{TOT}^{i} \geq 2.5ns \ and \ \langle\tau\rangle^{i} \geq 2.5ns$$

And we plotted in ***Figure 1*** of the main text the quantities $\log\frac{\tau_{TOT}^{i}}{\tau_0}$ versus $\log\frac{\langle\tau\rangle^{i}}{\tau_0}$ for every detected bond, with $\tau_0 = 2.5\ ns$ (this threshold was chosen in order to observe a meaningful number of hydrogen bonding events in both states). What emerged from this first analysis is that phosphorylated serines perform a higher number of hydrogen bonds, which generally have longer lifetime, and longer average lifetimes. Moreover, systems with phosphorylation show a higher variety of bonds, being capable of capturing different charged sidechains.

4. Distance distributions

In addition, we analysed the distance distribution between C_α^{SEP} and C_α of those residues involved in hydrogen bonds with highest total lifetime, and compared these results to the same $C_\alpha - C_\alpha$ pair in the WT (unphosphorylated) case. Results in ***Figure S1*** highlight that differences in hydrogen bonding are clearly reflected in distances between backbone atoms, propagating the local effect of hydrogen bonds also on backbone dynamics and overall loop conformations.

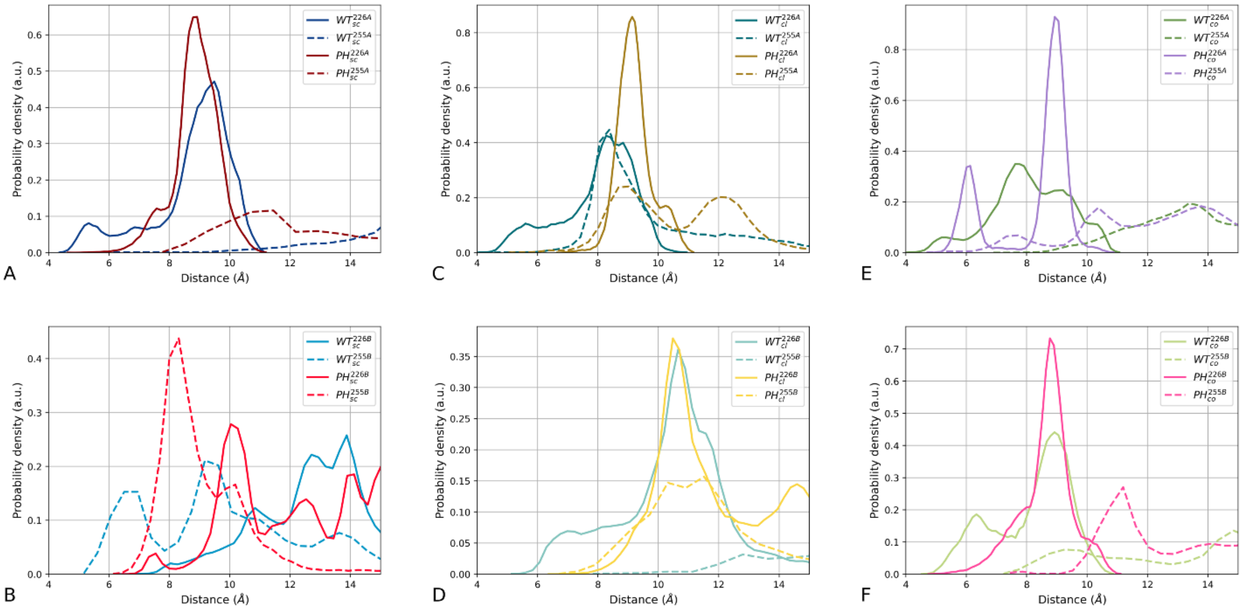

Figure S1 Distance distribution between (un)phosphorylated serine C_α atoms and C_α atoms of the Lys/Arg residues with the longest hydrogen-bonding lifetime in the phosphorylated states compared to the same WT distribution; dotted or solid line distinguish between the two serines in each monomer loop: **A)** Distance distribution values for monomer A loop serine residues in Semi-closed state in the WT or phosphorylated variants, **B)** Distance distribution values for monomer B loop serine residues in Semi-closed state in the WT or phosphorylated variants, **C)** Distance distribution values for monomer A loop serine residues in Closed state in the WT or phosphorylated variants, **D)** Distance distribution values for monomer B loop serine residues in the Closed state in the WT or phosphorylated variants, **E)** Distance distribution values for monomer A loop serine residues in Complex state in the WT or phosphorylated variants, **F)** Distance distribution values for monomer B loop serine residues in Complex state in the WT or phosphorylated variants.

5. Radial Distribution Function analysis

The enhanced presence of hydrogen bonds in the phosphorylated variants could highlight the capability of these systems to trap charged residues in different local energy minima, preventing loops from exploring their potential outermost reaches, and possibly reducing client scouting activity.

To better understand the trapping of positively charged residues, we analysed the radial distribution functions (RDFs) between $O_{\gamma}^{SER/SEP}$ and all outermost sidechain heavy atoms ($N_{\zeta}^{LYS}, C_{\zeta}^{ARG}$) interacting with serines during the entire trajectory. With respect to the previous analysis, this expands also to the neighbouring residues and not just to the directly hydrogen bonded residues (***Figure S1***). In all cases, phosphorylated serines showed increased RDFs areas with respect to Wild-Type variants. This reinforces the conclusion that phosphorylation traps charged residues, lowering their capability to move around and explore conformational space.

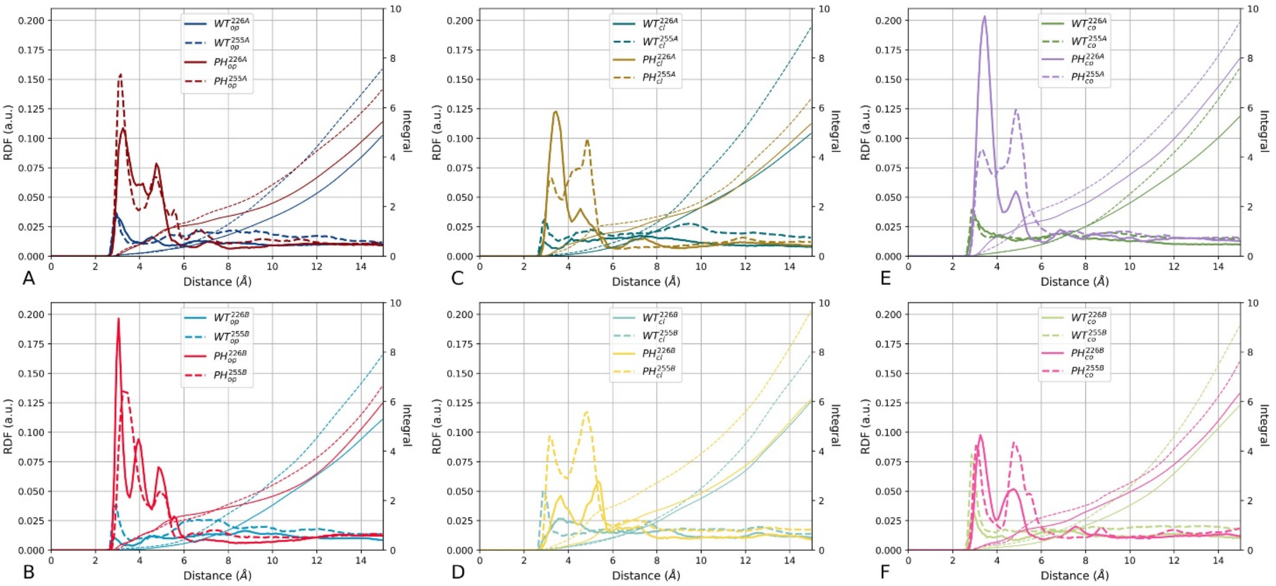

Figure S2 Radial distribution function of outermost heavy atoms of positive residues ($N_{\zeta}^{LYS}, C_{\zeta}^{ARG}$) with respect to $O_{\gamma}^{SER/SEP}$ in both the phosphorylated and Wild-Type variants; bolder lines represent the RDF itself, while thinner lines represent RDF integrals at increasing distances, dotted or solid lines distinguish between the two serines in each protomer loop: **A)** RDF and integral values for monomer A loop serine residues in Semi-closed state in the WT or phosphorylated variant, **B)** RDF and integral values for monomer B loop serine residues in Semi-closed state in the WT or phosphorylated variant, **C)** RDF and integral values for monomer A loop serine residues in Closed state in the WT or phosphorylated variant, **D)** RDF and integral values for monomer B loop serine residues in Closed state in the WT or phosphorylated variant, **E)** RDF and integral values for monomer A loop serine residues in Complex state in the WT or phosphorylated variant, **F)** RDF and integral values for monomer B loop serine residues in Complex state in the WT or phosphorylated variant.

6. *Sodium Ion Distribution*

A possible consequence of positive residues being trapped by phosphorylated serines is a higher exposure of negatively charged or polar residues to water and cations. Thus, considering sodium counterions as positively charged probes, we analysed the cumulative average number of Na^+ cations occurring within increasingly larger shells (+0.1 Å, from 0 to 20 Å) enveloping all loop atoms (***Figure S4***). The net charge difference between the WT and Phosphorylated system is -8 charges in total. However, Na^+ behaviour around loops changes across different states and also across the two monomers. Indeed, in the Semi-Closed state, in the Wild-Type variant, ions distributions around loops are comparable, while in the phosphorylated variant the two monomers have a net average difference of one unit charge in both shells (with protomer A being surrounded by more ions than protomer B).

In the Closed state, the trend is similar to the Semi-Closed state, with both WT protomers showing a small difference, while Phosphorylated protomers tend to have slightly different behaviour. In the complex state, in both cases, monomers display a markedly different behaviour, with protomer B being surrounded, overall, by a greater positive charge with respect to protomer A (a net difference of about +2). Interestingly, in this case the trend is opposite, with WT protomer B being surrounded by a larger number of cations with respect to protomer A. In the phosphorylated state the trend is reversed, with protomer B displaying a larger number of surrounding ions. This marked difference could suggest a weaker interaction between Hsp90 and the client when phosphorylation is present. Indeed, in the starting structure, protomer B of the chaperone is the one responsible for client interaction, thus, one would expect a lower available sampled volume and a reduced number of interactions with ions with respect to A loop, which is instead free to move around and reach the lumen. Overall, these tendencies could highlight an increased capability of phosphorylated Hsp90 to interact with positively charged protein residues, and also an increased interaction with clients.

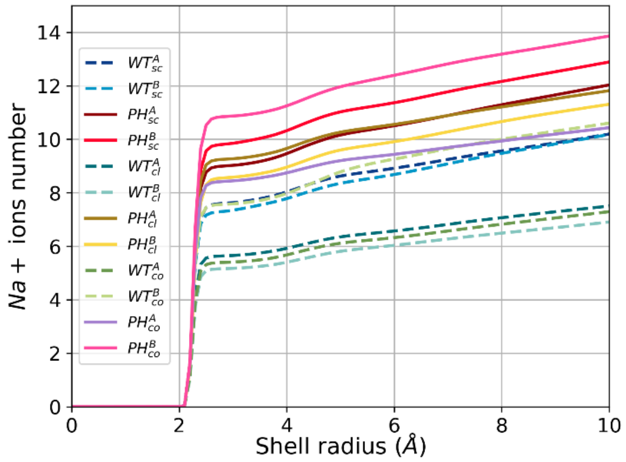

Figure S3 Average number of sodium cations found within a given shell radius from the charged loop.

7. Distance Fluctuation Analysis

Since Hsp90 is a highly interconnected and allosteric protein, we were also interested in capturing possible differences in global motions and network properties of structured domains of Hsp90. Thus, we started by comparing distance fluctuations matrices to gain insights on how the phosphorylation effects reverberate also to distal sites. Distance fluctuation analysis was developed to study how different distal sites in a protein or protein complex are allosterically connected, by calculating the variance of the distance between each pair of C_α atoms. Lower DF values correspond to lower fluctuation and high coordination, while higher fluctuations correspond to a lower degree of mechanical coordination. To better understand the differences between WT and phosphorylated system we calculated the ΔDF matrix, namely the subtraction of the WT matrix from the phosphorylated matrix [14-16]. This could help us in determining which regions are most affected by the phosphorylation, changing their allosteric coordination.

The ΔDF matrix of the Semi-Closed state displays a strongly altered dynamical behaviour, pointing out that phosphorylation indeed has a widespread effect also on structured domains. Regarding the intra protomer differences, in both protomers, phosphorylation induces a generalized loss of allosteric coordination (higher fluctuation), especially on the motion of the loop. Moreover, NTD_A loses coordination with the other structured domains (MiD_A and CTD_A). The same applies also in intra protomer communication pattern for protomer B, except that in this case phosphorylation increases loop coordination. Moreover, the mechanical coordination pattern appears to be modified also on inter protomer communication, with an overall increase in fluctuations after the phosphorylation. Despite the global trend of increased fluctuations, some regions appear to be less affected by phosphorylation, actually lowering their fluctuating motion. This can be due to the presence of longer-lived hydrogen bonds between phosphorylated serine residues and residues outside of the loop, which could serve as anchor points for loop mobility.

Moving to Closed state, the pattern remains similar to the Semi-closed state; indeed, in both monomers, NTD is showing a higher fluctuation with respect to Middle and C-Terminal Domains of the same monomers, while, on the other hand, both loops tend to increase their coordination with the rest of the complex. This could be due to the higher number of hydrogen bonds formed between the loops and the structured domain, with residues on NTD, MiD and CTD working as “trapping” residues that capture the loops and restrain their mobility. This remains true also in the inter monomer communication pattern, where NTDs lose coordination with CTDs, and inter loop coordination is again increased.

In the case of the Complex State, a clear pattern emerges, with the phosphorylated system being more prominently interconnected and rigidified from an allosteric point of view, with a widespread effect of stabilization performed by the phosphorylated sites. This effect propagates also to cochaperone proteins, which amplify the restraining effect played by modified serines.

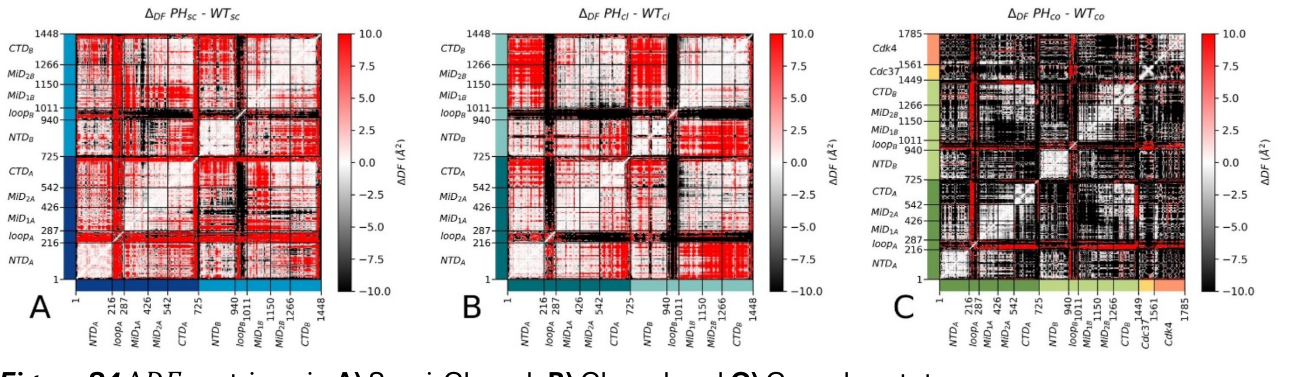

Figure S4 ΔDF matrices in **A)** Semi-Closed, **B)** Closed and **C)** Complex states.

8. Volume Explored by Loops

To further test this hypothesis and to study differences between conformations adopted by the charged loops, we utilized the *grid* command of *cpptraj*. In order to study and differentiate loop conformations, we first aligned frames of each trajectory to the first frame, with the alignment performed only on backbone atoms of secondary structure of Hsp90. From the aligned trajectory we calculated an average frame which was used as reference for a second realignment procedure, again on the backbone atoms of secondary structures Hsp90. Thus, we obtained a trajectory fixed on the resolved domains of Hsp90. Through the *grid* command we further built a cubic grid of points with 2.5 Å spacing and dimension 375 $\times$ 375 $\times$ 375 Å^3, cumulatively counting the number of frames in which a given loop atom (residues 216-278 in each protomer) was found to be adjacent to a grid point. We then plotted the volume occupied at least for the 25% of selected frames. Results are plotted in ***Figure 3*** in the main text.

In the open system loops tend to occupy almost the same region: in protomer A, the phosphorylated variant explores a wider region, interacting also with regions near the lumen, instead, the Wild-Type variant remains in a more globular conformation; in protomer B, the phosphorylated variant remains more globular and explores regions of space at larger distances with respect to the WT loop.

In the closed system, WT protomer A tends to explore regions closer to Hsp90 lumen, while the phosphorylated variant remains more globular. The behaviour of protomer B is similar in the two cases, with the phosphorylated loop occupying a slightly larger region in front of NTD_B.

In the complex system, phosphorylated protomer A explores a larger region of space, interacting also with the lumen, while the WT loop A stays in a more globular conformation. The B loop behaviour is similar, with the phosphorylated system exploring a smaller subspace region and also displaying a smaller surface interaction with the Cdk4 client.

9. Tensor Of Gyration (TOG)

To deepen our understanding of the global behaviour of Hsp90 under phosphorylated or Wild-Type conditions, we decided to apply dimensionality reduction with a method called TOG (Tensor Of Gyration), recently introduced [14]. Basically, this method is based on the identification of some key domains which can be approximated as ellipsoids. Then, by means of a selection based on DF_{score}, residues inside these domains are retained, and every ellipsoidal domain is further mapped to seven points, one of which is the center of mass of the domains, and the remaining 6 are two points for each axis (*x, y, z*) of the ellipsoid. This dimensionality reduction enables easier identification of global motions by means of a PCA analysis and of the calculation of Superposition and Alignment map [14].

In the case of Semi-Closed state, the superposition map suggests that the domains that are most affected by phosphorylation are the NTDs. Indeed, even after an optimal translation, the superposition of distance distributions between NTDs and other domains of the same protomers remains poor, highlighting a difference in the type of conformations explored during the dynamics. The alignment map gives a slightly more complete view, confirming the subtle nature of different motion of NTDs (in protomer A, NTD tends to stay closer to MiD and CTD, while in protomer B it tends to detach from them), which, however, display motions in the order of few tenths of Å. The higher effect of the phosphorylation is instead visible in the inter-protomer motion; the alignment map suggests that a global closure movement occurs after the perturbation, apart from NTD_A which tends to move farther apart from NTD_B (almost all distributions of pair distances needs to be positively translated after the phosphorylation to restore a movement distribution similar to the WT). Notwithstanding the global trend, the entity of the global displacement remains of 2 Å in terms of reduced points pairwise distance.

In the Closed state, the superposition map highlights that, even after the alignment procedure, the motion of the two complexes is not well comparable in all cases. The alignment map trend is similar to what is observed in the Semi-closed state, with protomer A slightly closing onto itself and protomer B slightly opening after the phosphorylation. The most visible effect is again that of a closing movement between $MiDs_A$ domains and protomer B, with NTD_A moving apart.

Complex state results align well with the Semi-Closed state; the superposition map highlights a different behaviour on different Hsp90 domains and also on the additional reduced points built on cochaperone and client. The alignment map again highlights the closure movement between MiD domains of both monomers after the phosphorylation. Instead, CTD_B domain displaces from all other domains. Regarding the cochaperone and client, Cdc37 stays closer to NTD domains in the phosphorylated case, while Cdk4 tends to stay closer to MiD_2s and CTDs in the phosphorylated case, suggesting higher interactions with Hsp90.

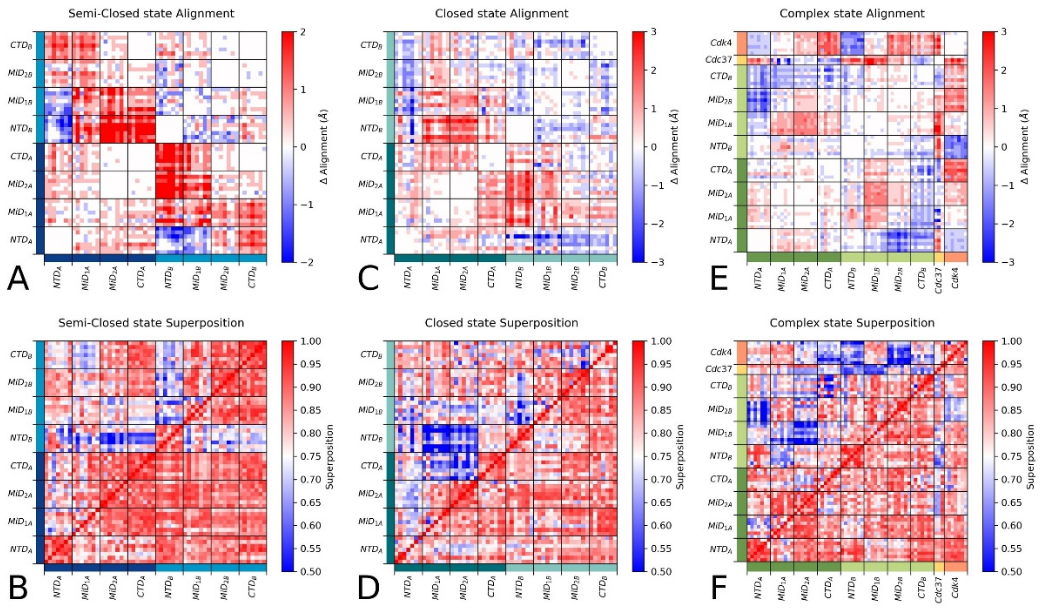

Figure S5 Alignment maps and Superposition maps for **A,B)** Semi-Closed, **C,D)** Closed and **E,F)** Complex states. In the Alignment map blue means low superposition between distance distribution, suggesting a substantial difference in motions of the points in the two states, while red means a high superposition. In Superposition map the colour refers to the translation needed to have the maximum superposition between distance distribution, for example blue values in the **A)** matrix mean that the phosphorylated variant of the Semi-Closed state needs to be translated back to match Wild-Type variant behaviour, while red values mean the reverse.

Once we identified these main movements and differences between phosphorylated and Wild-Type systems, we calculated the PCA of reduced TOG trajectories to map them onto the structure, projecting the first PC on the reduced points. In the Semi-closed complex, the main difference is located in the direction of NTD_A movement. While in the WT variant directions of motion point outwards, in the phosphorylated case they are more dispersed and do not point out in a unique direction. Another difference is located in $MiD_{1,2B}$ domains, that in the Wild-Type variant they tend to move outwards, while no major movement is observed in the phosphorylated case.
The Closed complex features different movements depending on the variant. While the major movement of WT corresponds to a partial closure at CTDs, that tend to close upon one another, in the phosphorylated case, movements at CTDs are quite dispersed, and the major movement is located at NTD_A, which tends to move towards the lumen.
Regarding the Complex state, we studied the movements related to Hsp90 complex and Hsp90:Cdk4 complex, since Cdc37 is composed of a double helical domain which tends to perform a spanning movement that encodes the majority of the fluctuations, and PCA returns a movement mainly located around that domain, which is not relevant. Analysis focused on Hsp90 highlights that the first principal component is that of a generalized twisting motion around the lumen, that in the phosphorylated system seems to have a larger entity.

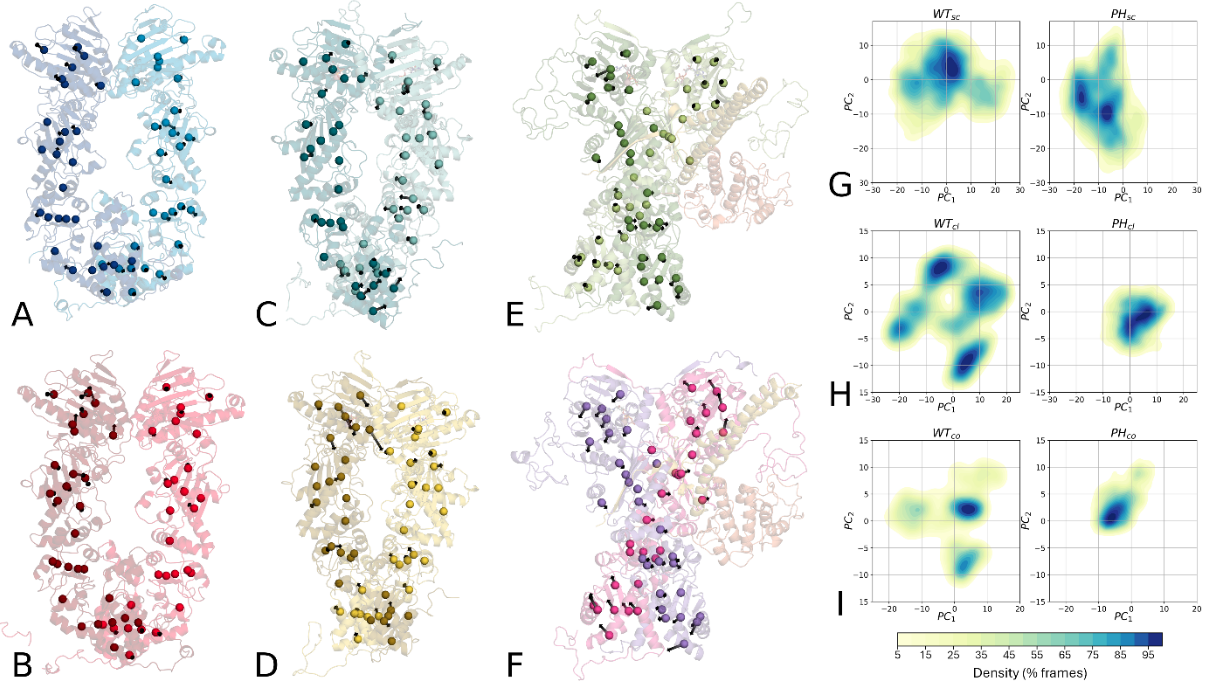

Figure S6 First principal component of TOG trajectories in **A)** WT variant, Semi-Closed state, **B)** Phosphorylated variant, Semi-Closed state, **C)** WT variant, Closed state, **D)** Phosphorylated variant, Closed state, **E)** WT variant, Complex state, **F)** Phosphorylated variant, complex state, and Kernel Density Estimate plots of the first two principal components for the phosphorylated (right) and WT variants (left) in **G)** the Semi-Closed state, **H)** the Closed state, **I)** the Complex state.

Despite the low visual similarity of principal motions, we projected frames of phosphorylated systems onto the first two principal components of WT systems. In all cases a lower portion of principal component space is explored by phosphorylated variants, enhancing the idea that phosphorylation changes the capability of Hsp90 machinery to sample the phase space. When trying to couple the principal component directions, we attempted to identify two physical variables that could serve as collective variables to describe the motion of different Hsp90 domains. Chosen variables were: (1) the angle between the vectors connecting the centers of mass of NTDs and CTDs, corresponding to a “wrapping” variable that explains the rotation of one monomer onto the other; and (2) reduced lumen volume, calculated with the triangulation of different points facing the lumen space (see ***Figure 3*** of the main text).

This combination of collective variables better exalts the differences induced by phosphorylation in the conformational cycle of Hsp90. In the Semi-closed state, the phosphorylated variant displays a lower average loop volume and a more closed NTDs conformation (large angles correspond to large twisting motion of NTDs with respect to CTDs, ***Figure 3C*** of the main text). In the Closed conformation, the similarity between the two states increases, with the lumen of the phosphorylated variant being slightly more open and with similar NTD-CTD closing angle (***Figure 3D*** of the main text). Complex state CV distribution highlights the more compact nature of phosphorylated variant, which has a single explored basin at reduced lumen volume, while in the WT case Hsp90 experiences a wider motion which leads to partial opening of the lumen (with respect to phosphorylated variant) together with a larger angular movement between NTDs and CTDs ***Figure 3E*** of the main text.

References

1. Schrödinger, L. The Pymol Molecular Graphics System, Version 1.8. www.schrodinger.com
2. Sali, A.; Blundell, T. L. Comparative Protein Modeling by Satisfaction of Spatial Restraints. *Journal of Molecular Biology* **1993**, *234*, 779-815.
3. Fiser, A.; Do, R. K.; Sali, A. Modeling of Loops in Protein Structures. *Prot. Sci.* **2000**, *9*.
4. Maier, J. A.; Martinez, C.; Kasavajhala, K.; Wickstrom, L.; Hauser, K. E.; Simmerling, C. Ff14sb: Improving the Accuracy of Protein Side Chain and Backbone Parameters from Ff99sb. *Journal of Chemical Theory and Computation* **2015**, *11*, 3696-3713.
5. Jorgensen, W. L.; Chandrasekhar, J.; Madura, J.; Impey, R. W.; Klein, M. L. Comparison of Simple Potential Functions for Simulating Liquid Water. *J. Chem. Phys.* **1983**, *79*, 926-935.
6. Joung, I. S.; Cheatham, T. E. Determination of Alkali and Halide Monovalent Ion Parameters for Use in Explicitly Solvated Biomolecular Simulations. *J. Phys. Chem. B* **2008**, *112*, 9020-9041.
7. Allnér, O.; Nilsson, L.; Villa, A. Magnesium Ion–Water Coordination and Exchange in Biomolecular Simulations. *Journal of Chemical Theory and Computation* **2012**, *8*, 1493-1502.
8. Case, D. A.; Aktulga, H. M.; Belfon, K.; Cerutti, D. S.; Cisneros, G. A.; Cruzeiro, V. W. D.; Forouzesh, N.; Giese, T. J.; Götz, A. W.; Gohlke, H.; Izadi, S.; Kasavajhala, K.; Kaymak, M. C.; King, E.; Kurtzman, T.; Lee, T.-S.; Li, P.; Liu, J.; Luchko, T.; Luo, R.; Manathunga, M.; Machado, M. R.; Nguyen, H. M.; O’Hearn, K. A.; Onufriev, A. V.; Pan, F.; Pantano, S.; Qi, R.; Rahnamoun, A.; Risheh, A.; Schott-Verdugo, S.; Shajan, A.; Swails, J.; Wang, J.; Wei, H.; Wu, X.; Wu, Y.; Zhang, S.; Zhao, S.; Zhu, Q.; Cheatham, T. E., III; Roe, D. R.; Roitberg, A.; Simmerling, C.; York, D. M.; Nagan, M. C.; Merz, K. M., Jr. Ambertools. *Journal of Chemical Information and Modeling* **2023**, *63*, 6183-6191.
9. Feller, S. E.; Zhang, Y.; Pastor, R. W.; Brooks, B. B. Constant Pressure Molecular Dynamics Simulation: The Langevin Piston Method. *J. Chem. Phys.* **1995**, *103*, 4613-4621.
10. Bussi, G.; Donadio, D.; Parrinello, M. Canonical Sampling through Velocity Rescaling. *The Journal of Chemical Physics* **2007**, *126*, 014101.
11. Miyamoto, S.; Kollman, P. A. Settle: An Analytical Version of the Shake and Rattle Algorithms for Rigid Water Models. *J. Comp. Chem.* **1992**, *13*, 952-962.
12. Berendsen, H. J. C.; Postma, J. P. M.; van Gunsteren, W. F.; Di Nola, A.; Haak, J. R. Molecular Dynamics with Coupling to an External Bath. *J. Chem. Phys.* **1984**, *81*, 3684-3690.
13. Darden, T.; York, D.; Pedersen, L. Particle Mesh Ewald: An N-Log(N) Method for Ewald Sums in Large Systems. *J. Chem. Phys.* **1993**, *98*.

14. Magni, A.; Bonollo, G.; Trèves, G.; Frigerio, F.; Cinquini, F.; Pavoni, S.; Oliveira, A. S. F.; Mulholland, A. J.; Serapian, S. A.; Colombo, G. Ligand-Driven Modulation of Chaperone–Cochaperone Networks Shapes Proteostasis Outcomes. *Protein Science* **2026**, *35*, e70543.
15. Moroni, E.; Agard, D. A.; Colombo, G. The Structural Asymmetry of Mitochondrial Hsp90 (Trap1) Determines Fine Tuning of Functional Dynamics. *J. Chem. Theory Comput.* **2018**, *14*, 1033-1044.
16. Morra, G.; Potestio, R.; Micheletti, C.; Colombo, G. Corresponding Functional Dynamics across the Hsp90 Chaperone Family: Insights from a Multiscale Analysis of Md Simulations. *Plos Comput. Biol.* **2012**, *8*, e1002433.
